# Obesity in prematurely born children and adolescents: follow up in pediatric clinic

**DOI:** 10.1186/1475-2891-12-150

**Published:** 2013-11-19

**Authors:** Tetyana L Vasylyeva, Apurv Barche, Sudha P Chennasamudram, Christopher Sheehan, Ruchi Singh, Michael E Okogbo

**Affiliations:** 1 Department of Pediatrics, Texas Tech University Health Sciences Center, Amarillo, Texas, USA; 2 Department of Pediatrics, Scott & White/Texas A&M College of Medicine Temple, Texas, USA

**Keywords:** Obesity, Prematurity, Low birth weight, Large for gestational age, Postnatal care

## Abstract

**Background:**

Understanding associated risk for obesity is a prerequisite to develop early life interventions to arrest the increasing epidemic of metabolic syndrome and obesity among preterm born children and adolescents.

**Findings:**

A retrospective review of 160 charts was conducted to determine the associated risk of being obese during childhood and adolescent period in preterm children. Birth weight, gestational age, weight gain, demographics, maternal health, socioeconomics, and clinical factors during early neonatal life were evaluated. The number of obese children increased with age and was observed more in the adolescent population. Obese children were significantly heavier at age 24 months old compared to their peers (*p* = 0.001). Analysis of associated risk for maternal demographics, maternal age, maternal marital status or race, prenatal factors, maternal substance abuse or diabetes, neonatal factors, weight for gestational age or birth weight did not show any statistically significant risk for future obesity. Duration of gestational age (OR 1.6; *p* = 0.017) and heavier birth weight (OR 3.2; *p* = 0.001) were associated with risk of obesity.

**Conclusion:**

Among preterm born babies in the study, the highest risk of developing excessive weight during childhood and adolescent periods are babies born at more advanced gestational age. Strong positive association was found between birth weight and body weight in childhood. By 24 months old, there was a distinguished group of toddlers, who were heavier than their peers and remained with excessive weight as they got older. Primary care pediatricians should draw attention to premature babies, overweight infants and toddlers.

## Background

Obesity is the main focus of healthcare providers and healthcare policy due to a sustained increased in prevalence over the last decade [1-5]. A consistent body of evidence now demonstrates that being overweight or obese in childhood and adolescence has adverse consequences on overall health and leads to premature mortality and increased physical morbidity in adulthood [6].

Low birth weight (LBW) premature infants demonstrate growth patterns in the early years of their life (catch up growth) which differ from those of large for gestational age (LGA) term and preterm peers, who do not experience that steep weight gain [7,8]. Very low birth weight (VLBW) children who demonstrate excessive weight gain during infancy had a greater likelihood of adult obesity, cardiovascular disease, and diabetes [8]. With incredible improvement of management and survival of premature babies, their life-long management has become an important issue in the general pediatric clinic [9].

Numerous clinical and experimental studies have confirmed that early developmental influences can lead to cardiovascular, pulmonary, metabolic, and psychological diseases during adulthood regardless of birth weight [9]. Children small for gestational age may be predisposed to metabolic abnormalities upon exposure to postnatal environmental risk factors such as, low physical activity and/or high-energy intake [10]. Primary care pediatricians and registered dietitians have a leading role in understanding the associated risk factors and initiating early intervention including, diet modification and diet regimen adjustments to provide essential care for the lifelong well-being of premature children.

The objective of the study was to identify significant risk factors in prematurely born children leading to the development of obesity during late childhood and adolescence periods.

## Methods

All data was abstracted from the charts of general pediatric clinic records for children and adolescents from 10 to 21 years old, who were born at 37 or fewer weeks of gestational age. The study protocol was approved by the Amarillo TTUHSC Institutional Review Board. Charts were pulled by electronic records using ICD-9 code for prematurity. Demographic, maternal, family, neonatal, and postnatal data were abstracted. Maternal and family history included: previous premature births, race, marital status, prenatal care, health insurance, maternal age, maternal substance abuse, maternal illnesses (such as genital and urinary tract infections, preeclampsia, hypertension, and diabetes mellitus), and previous multiple gestations. History during the neonatal period included severe illness, respiratory distress syndrome, umbilical vein or artery catheter use, oxygen therapy, intracerebral and intra ventricular hemorrhage, bacterial infections, and metabolic diseases, such as hypoglycemia and jaundice. The child’s postnatal data included type of feeding, major illnesses, blood pressure, weight, and height. Body Mass Index (BMI) was calculated as the patient's weight (kg)/height^2^ (m). Obesity was defined as a BMI at or above the 95th percentile for children of the same age and sex [11]. Term “excessive weight” also has been used to indicate “obese” children. Overweight children with BMI at or above the 85th percentile and lower than the 95th percentile for children of the same age and sex were excluded from the study. We used CDC 2000 BMI charts for children older than 2 year old and weight/stature charts for the younger age [12].

Cases were classified as extremely low birth weight (ELBW) if less than 1000 g, very low birth weight (VLBW) if birth weight was less than 1500 g, and low birth weight (LBW) if less than 2500 g. Small for gestational age (SGA) was defined as a birth weight less than the 10th percentile for the baby's gestational age group, and large for gestational age (LGA) was defined as a birth weight greater than the 90th percentile for the infant’s gestational age group. All charts had multiple visits records and all visits data were abstracted. For children older than one year of age the majority of the charts did not have a detail nutrition description. For hypertension assessment at least three recorded elevated blood pressure measurements have been used.

Statistical analysis was performed using Stata, Version 10 to summarize descriptive data and to calculate odds ratios to estimate the association between the prevalence of obesity and each variable of interest. To compare weight gains before 24 months in obese and non-obese groups the t-test was used. Correlation between weight gains during the first three years of life and reported BMI between 10 and 21 years of age was done. Computation of Mann–Whitney U tests for obese subjects (BMI at the 95th percentile or higher) to weight gains was also analyzed.

## Results

One hundred sixty eligible preterm cases were abstracted from the clinic charts. Among the sample, 18 babies were ELBW (gestational age (GA) ranged 27.4 ± 2.7), 19 were VLBW (GA ranged 30.7 ± 1.9), 87 were LBW (GA ranged 33.6 ± 2.1) and 36 were NBW (GA ranged 35.5 ± 1.3). Among VLBW babies average BW was 821 ± 138 grams, among LBW - 1983 ± 262 grams, NBW 2869 ± 418 grams. There were 59% male and 41% female.

**Figure 1:**
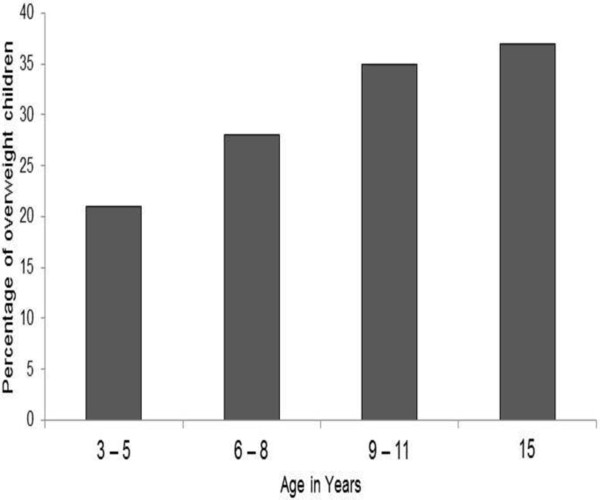
Number of obese children (%) among pre-term born pediatric population.

The number of obese children increased with age and this trend was observed more in the adolescent population (Figure 1). Analysis of the association between BMI and the multiple parameters mentioned in the methods section showed some associations (Table 1). Traditional preterm delivery risk factors such as maternal age, ethnicity, social status, smoking and hypertension did not show significant association with offspring’s childhood obesity in the study.

**Table 1:** Age-adjusted odds ratios of demographic, medical history and clinical factors in obese children at any time before age 21 within the cohort of premature born babies

	OR*	**Lower †CI**	**Upper CI**	***p*-value**
**Maternal demographics**				
Age (odds per 1 SD age increase)	1	0.96	1.1	0.341
**Race (compared with odds among Hispanic **_**(n = 49)**_**)**				
White _(n = 51)_	0.76	0.3	1.9	0.560
Black _(n = 11)_	0.61	0.11	3.2	0.570
**Marital status (comparison of odds with being single **_**(n = 42)**_**)**				
Married _(n = 65)_	1.05	0.46	2.41	0.900
Divorced _(n = 6)_	1.8	0.32	11	0.491
Married _(n = 83)_ (*v.* never-married _(n = 77)_)	0.64	0.22	1.9	0.416
Currently single _(n = 42)_ (*v.* no single _( n= 118)_)	1.4	0.48	4.3	0.516
**Maternal health status**				
Smoking _(n = 32)_ (*v.* non-smoking_(n = 128)_)	0.76	0.29	1.98	0.58
Hypertension _(n = 20)_ (*v.* no reported hypertension _(n = 19)_)	0.55	0.14	2.1	0.391
Diabetes _(n = 47)_ (*v*. no diabetes _(n = 113)_)	1.13	0.41	3.1	0.810
**Neonatal metrics**				
Length of gestation (days) (risk odds per 1 SD increase in length of gestation)	1.6	1.1	2.4	**0.017**
**Weight according gestational age (comparison of odds among average, AGA **_**(n = 124)**_**)**				
Small for gestational age, SGA _(n = 23)_	0.47	0.13	169	0.250
Large for gestational age, LGA _(n = 13)_	1.4	0.40	4.84	0.600
**Categorized gestational weight (comparison of odds with among normal BW (NBW **_**(n = 36)**_**)**				
Extremely low birth weight, ELBW _(n = 18)_	0.17	0.02	1.52	0.114
Very low birth weight, VLBW _(n = 19)_	0.10	0.28	3.53	1.00
Low birth weight, LBW _(n = 87)_	1.03	0.42	2.5	0.950
Birth weight (odds per 1 SD of increase BMI)	3.2	1.7	5.7	**0.001**
Gender (male _n = 95_*v.* female _n = 65_)	1.9	0.68	5.2	0.225
**Feeding status (comparison of odds among the exclusively breast fed **_**(n = 15)**_**)**				
Formula _(n = 67)_	1.6	0.03	1	0.056
Combination breast/formula _(n = 67)_	0.44	0.07	2.8	0.387
**Catheters in NICU**				
Umbilical artery catheter _(n = 27)_	0.92	0.28	3.1	0.89
Umbilical venous catheter _(n = 41)_	0.6	0.19	1.9	0.379
**Clinical parameters**				
Age (odds per 1 SD age increase)	3.4	2.4	4.6	**0.001**
Height (odds per 1 SD hight increase)	12	2.6	53	**0.001**
Systolic blood pressure (odds per 1 SD increase of BMI)	2.4	1.7	3.3	**0.001**
Diastolic blood pressure (odds per 1 SD increase of BMI)	1.4	1.1	1.9	**0.021**

*OR: odds ratio comparisons as noted; some are binary comparisons, some to a baseline for categorical variables. The table represent partial list of evaluated variables. †Cl: confidence interval, SD: standard deviation, BMI: body mass index.

Strong positive association was found between birth weight increase *vs*. odds per 1 SD of body weight increase in childhood (OR 3.2; *p* = 0.001). Increased gestational age in preterm babies was also associated with excessive weight during maturation in childhood (OR 1.6; *p* = 0.017). Again strong associated risks were noticed between weight and elevated blood pressure, affecting systolic (OR 2.4; *p* = 0.001) more so than diastolic (OR 1.4; *p* = 0.021).

**Figure 2:**
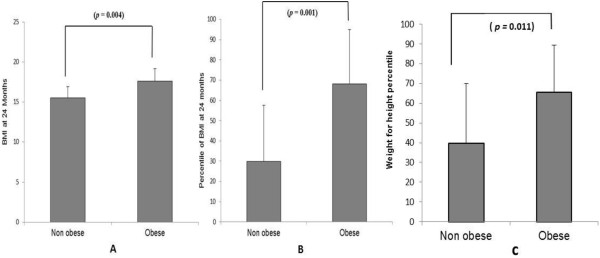
**Obese adolescent showed significantly higher weight at the age of 24 months as compared with peers who were not obese later in childhood.** Graph **(A)** is a comparison of Body Mass Index (BMI), graph **(B)** is a comparison of percentile of BMIs and graph **(C)** is a comparison of weight for height percentile.

Computation of correlations between weight gains during the first three years of life and reported BMI between 10 and 21 years of age showed persistent, although not strong (max 0.45), positive correlation. Obese adolescents showed significantly higher weight at the age of 24 months as compared with peers who did not have excessive weight later in childhood (*p* = 0.001) (Figure 2).

## Discussion

Identification of early-life risk factors for developing obesity in prematurely born children is essential for development of early prevention interventions to reduce obesity. The postnatal period is a critical time when nutrition may predispose a child to lifelong metabolic disturbance and obesity. For LBW, premature, and small-for-gestational-age infants more research is needed to determine optimum nutrition needs [13].

Obesity, type 2 diabetes mellitus, hypertension, coronary artery disease and stroke might be the health consequences of being born SGA [1,14]. Reported earlier was the strong association between elevated blood pressure and body weight in preterm born children [15]. While doing post-hoc analysis it became apparent that there may be associative risks between systolic and diastolic blood pressure per 1 SD increase of BMI and confirmed the previous finding of strong association. Both hypertension and obesity are factors strongly associated with morbidity and mortality later in life. The fact that these two risk factors are observed and strongly associated in prematurely born children should rise vigilant of primary care physicians toward intensive preventive measurements in this population.

The search for associations between the development of excessive weight with numerous pre-, peri-, and post-natal variables in the study showed that preterm born children, who have a longer GA and higher BW were more at risk to become obese during childhood adolescent periods. Gaskins *et al.* also noticed that higher birth weight predicts childhood obesity in preterm infants [16]. The mechanism is still not completely understood, but may involve either altered appetite regulation or insulin secretion [17-19] and even hypothalamic inflammation [20]. Maternal over-nutrition has been shown deleterious to the health of offspring and could result in a phenotype of the offspring that is characteristic of metabolic syndrome [21]. Epigenetic factors have been proven to be significant in the etiopathogenesis of obesity of preterm children [22,23] and represent gene regulation through environmental impact.

The “postnatal accelerated growth” hypothesis was proposed to explain the association between faster, early postnatal growth and surrogate endpoints in childhood and adolescence indicative of metabolic and cardiovascular risks based on follow-ups of preterm infants in two early neonatal feeding/nutritional intervention trials [24,25]. A suggestion was made that early postnatal catch-up growth, rather than prematurity per se, leads to programming insulin resistance and related disorders [25]. At the same time, most preterm infants, especially those born very preterm with ELBW, not fed sufficient amounts of nutrients to produce normal fetal rates of growth after birth, end up growth-restricted during their hospital period [26]. Data from the other group do not support the idea of increased adiposity in preterm born babies fed with a nutrient-enriched formula after hospital discharge [27]. Studies suggested that a 'window of opportunity' existed after hospital discharge and that better growth between reaching the tem status period and 2–3 months corrected age was correlated with better development [27]. Animal experiments have shown that among low-birth weight male mice, neonatal catch-up growth normalized neurobehavioral and cardiovascular phenotypes, but led to insulin resistance and high fat diet-induced diabetes [28]. Resent study also showed that BMI gained over the 18 months after term had been associated with later risk of a child being overweight or obesity, but with low evidence of a benefit for IQ [29]. Thus, a nutritional goal for the premature infant with growth failure remains a source of controversy.

The data confirmed that overfeeding in the early stages of childhood is critical to excessive weight gain and obese children had significantly higher weight at age 24 months old compared with peers, who did not have excessive weight at toddler age. This finding was in agreement with the previous finding that children, who show early and complete growth recovery, could be at a higher risk for the occurrence of metabolic syndrome in adulthood [30]. The significant finding is that more mature, although preterm babies had a tendency for accelerated postnatal growth.

Although more focused research is needed to address this issue, a suggestion would be to reassess nutritional habits of all prematurely born children as soon as they achieve growth parameters of their peers during their well child checks. A nutrition model of preterm born children and adolescents with risk factors should be developed.

## Conclusion

Among preterm born babies in this study, the highest risk of developing obesity during childhood and adolescent period are babies born at more advanced gestational age. Strong positive association was found between birth weight and body weight in childhood. By 24 months old there was a distinguished group of toddlers, who were heavier than their peers and remained with excessive weight with age. Primary care pediatricians should draw attention to premature babies, overweight and obese infants and toddlers. This is a phase where well child checks are more frequent and education regarding adequate nutrition is essential. Timely referral to registered dietitians might help those children, who demonstrate excessive body weight gain during two consecutive well child visits in spite of dietary recommendations from primary health care providers.

## Abbreviations

LBW: Low birth weight; LGA: Large for gestational age; VLBW: Very low birth weight; HTN: Hypertension; SD: Standard deviation; BMI: Basic metabolic index; ELBW: Extremely low birth weight; SGA: Small for gestational age; GA: Gestational age; NBW: Normal birth weight; BW: Birth weight; OR: Odds ratio; CI: Confidence interval.

## Competing interests

The authors declare that they have no competing interest.

## Authors’ contributions

TLV designed the research and wrote the paper; CS did primary data collection and organization; AB, SPC and RS organized and analyzed the data; MEO provided consultation and pediatric charts for the research. All authors read and approved the final manuscript.
